# Measurement Properties and Implementation of a Checklist to Assess Leadership Skills during Interdisciplinary Rounds in the Intensive Care Unit

**DOI:** 10.1155/2015/951924

**Published:** 2015-01-29

**Authors:** Elsbeth C. M. Ten Have, Raoul E. Nap, Jaap E. Tulleken

**Affiliations:** ^1^Directorate of Medical Affairs, Quality and Safety, University Medical Center Groningen, University of Groningen, P.O. Box 30,001, 9700 LB Groningen, Netherlands; ^2^Department of Critical Care, University Medical Center Groningen, University of Groningen, P.O. Box 30,001, 9700 LB Groningen, Netherlands

## Abstract

The implementation of interdisciplinary teams in the intensive care unit (ICU) has focused attention on leadership behavior. A daily recurrent situation in ICUs in which both leadership behavior and interdisciplinary teamwork are integrated concerns the interdisciplinary rounds (IDRs). Although IDRs are recommended to provide optimal interdisciplinary and patient-centered care, there are no checklists available for leading physicians. We tested the measurement properties and implementation of a checklist to assess the quality of leadership skills in interdisciplinary rounds. The measurement properties of the checklist, which included 10 essential quality indicators, were tested for interrater reliability and internal consistency and by factor analysis. The interrater reliability among 3 raters was good (*κ*, 0.85) and the internal consistency was acceptable (*α*, 0.74). Factor analysis showed all factor loadings on 1 domain (>0.65). The checklist was further implemented during videotaped IDRs which were led by senior physicians and in which 99 patients were discussed. Implementation of the checklist showed a wide range of “no” and “yes” scores among the senior physicians. These results may underline the need for such a checklist to ensure tasks are synchronized within the team.

## 1. Introduction

The intensive care unit (ICU) is characterized by life-threatening and time-critical conditions which require the synchronized and collaborative efforts of professionals of several disciplines [1, 2]. Recent studies concerning optimal team ICU care mention the importance of interdisciplinary rounds (IDRs). IDRs are associated with improved patient outcomes, reductions in preventable harm, and fewer conflicts among team members [3–9]. Therefore, the Society of Critical Care Medicine has endorsed daily IDRs, which are defined as rounds where the appropriate plan of care is agreed on, understood, and executed as planned by all care providers [3, 10]. Although there is no ambiguity about the goal of IDRs, the execution varies because IDRs are complicated by factors including limited time, multiple targets, patient instability, highly technical therapies, and varied responsibilities of different care providers [5, 9, 11–14].

Studies concerning interdisciplinary teams in the ICU showed that the behavior of senior physicians seems to significantly influence the behavior of other team members because of the hierarchic nature [8, 9, 15–17]. These findings have brought attention to the relevance of leadership. Leadership skills are crucially important for determining the extent to which interdisciplinary teams provide coordinated and safe patient care [6, 8, 18].

During IDRs, leading physicians have difficulty determining whether or not important aspects of the patients' plan of care are communicated effectively. Several studies have described such problems [8, 19, 20]. Recent studies revealed that checklists may be useful to structure the interdisciplinary communication process in complex and dynamic situations such as IDRs in the ICU, but little information is available about checklists which evaluate leadership skills during IDRs [21–23].

We performed a study that tested the measurement properties and implementation of a checklist aimed at assessing the quality of leadership skills while leading IDRs in the ICU.

## 2. Methods

### 2.1. Study Setting

This study was performed in 4 adult ICUs at the University Medical Center in Groningen, the Netherlands. In sum, these ICUs (thoracic, medical, surgical, and neurologic) admit approximately 3,000 patients per year. In all 4 ICUs, daily IDRs were organized separately from morning rounds and change-of-shift reports. At the IDRs, specialists shared information, addressed patient problems, and planned and evaluated treatments [11, 24]. In a typical IDR starting at 11:00 AM, the care plans of approximately 12 patients were discussed over a 2-hour time period. Senior physicians (intensivists) led the sessions, junior physicians gave clinical patient presentations, and bedside nurses and consultants gave additional current and appropriate information. The presence of specialist consultants varied with each patient and included surgeons, respiratory specialists, nephrologists, or neurologists.

The study period ranged from July 2009 to May 2011. For the present study, we analyzed data from 10 IDRs led by 10 experienced intensivists. Before the IDR started, a video camera was placed in the corner of the meeting room to enable the rating of all participants. At the end of the IDR, the video camera was removed.

The Medical Ethical Testing Committee of the University of Groningen waived Institutional Research Board approval for videotaping IDRs in the ICU because of the observational design of the study and because of the fact that staff members (not patients) were the study subjects.

### 2.2. Participants

All participants in the IDRs were informed during ward and staff meetings about the videotaping. The intensivists (9 men and 1 woman) each had 3–20 years of clinical experience as certified intensivists. In order to lead IDRs, intensivists were previously trained by role modeling colleagues or other senior physicians.

The intensivists each volunteered to being videotaped while leading one IDR, so the schedule for videotaping IDRs was adapted to their roster. Their performance was individually assessed and discussed in reference to the checklist. Anonymity of all participants was assured.

### 2.3. Development of the Checklist

The development of the checklist was based on (1) the principles applied in human factors engineering as described by Winters et al. and (2) the Interdisciplinary Rounds Assessment Scale [22]. The IDR Assessment Scale was previously developed to assess the quality of performed IDRs in the ICU and was described in detail elsewhere [24]. The assessment of leadership skills was not incorporated in this scale.

To test this IDR Assessment Scale, an exploratory factor analysis was performed using the rotation method (Varimax with Kaiser normalization) [24]. The exploratory factor analysis revealed a solution with the minimum of three domains [25]. A confirmatory factor analysis subdivided the 19 quality indicators into 10 essential (with factor loadings on the first domain > 0.65) and 9 supportive indicators.

Because confirmation of a well performed IDR in the ICU was reached when the 10 essential quality indicators were rated as “yes” or “not applicable,” we extracted these 10 essential quality indicators for further testing [24].

We compared the essential quality indicators of the tool with results of a literature search about leadership in the ICU [8, 26–29]. The search showed that perceived strong leadership focuses on an open atmosphere and support for team members by developing a common perspective on the goals, defining boundaries, and managing expectations, contributed significantly to good patient outcome, such as reduced length of stay [12, 16, 30]. Leadership was defined as “the process of influencing others to understand and agree about what needs to be done and how to do it, and facilitating individual and collective efforts to accomplish shared objectives” [31]. From the perspective of the team process, leadership includes a clear understanding of joint responsibilities, along with continuous active cross-checking, to prevent key activities from escaping attention [15, 16]. In addition, the indicators were checked by asking critical care physicians, nurses, and trainers for suggestions to reduce ambiguity [22].

As a result, no additional indicators were considered useful to lead IDRs [32]. Leadership components were considered to be reflected in the 10 essential quality indicators which we combined into the checklist (Table 1).

### 2.4. Implementation of the Checklist

Implementation of the checklist was realized by rating (in real life and by analyzing videotapes) whether or not the 10 leading intensivists demonstrated (non)verbal behavior which corresponded with the respective quality indicators during 99 individual patient presentations. A 3-point scale was used to classify this behavior: (1) no (the behavior was not observed; 1 point), (2) doubt/inconsistent (verbalizations or behaviors were inconsistent with the quality indicator; 2 points), or (3) yes (the behavior was clearly observed and was consistent with the quality indicator; 3 points).

Some items had a “not applicable” option if the indicator could not be rated. The “not applicable” option was incorporated because indicators such as diagnostic plan discussed, long term interventions discussed, and patient greatest risk discussed may not be applicable in case of end-of-life palliative care consultation or discharge from the ICU. The “not applicable” option was also incorporated for indicators which were related to junior physicians, ICU nurses, and/or specialist consultants, to facilitate implementation of the checklist to ICUs which were structured in different ways.

### 2.5. Training of Raters for Assessment

There were 3 raters, including 1 senior physician, 1 ICU nurse, and 1 author (communication trainer and nurse), who were trained by assessing 9 videotaped patient presentations led by different intensivists. Responses were checked manually to confirm definitions were applied uniformly and by testing interrater reliability (definitions extracted from the manual were shown in Table 1). When the interrater reliability was ≥0.70, the training was considered effective and the 3 raters were allowed to rate other patient presentations. The quality of the individually tested patient presentations was checked through random testing of patient presentations by another rater to determine whether interrater reliability was ≥0.70 [33].

### 2.6. Statistical Analysis

The measurement properties of the checklist were tested using interrater reliability and internal consistency and by factor analysis [34]. Interrater reliability was tested by 3 raters who examined the indicators in 9 randomly selected patient presentations [33, 35, 36]. Internal consistency was measured with Cronbach *α*. A confirmatory factor analysis was previously performed using the rotation method (Varimax with Kaiser normalization) [24].

To evaluate the leading skills of the intensivists, we used the *χ*² test (chi-square test) [37]. This test uses descriptive statistics of data and compares the range of frequencies of each essential quality indicator by each physician. The hypothesized standard is the fact that “all 10 essential quality indicators are rated with “yes” or “not applicable” in 90% of each patient discussed during the IDR.” Significant outcomes imply deviance from the hypothesized standard, while nonsignificant outcomes imply a failure to reject the null hypothesis.

## 3. Results

The interrater reliability of the checklist among the 3 raters was good (*κ*, 0.85). To decrease potential bias from shared understanding of the developed methods another 20 patient presentations were corroborated by an additional independent nonmedical rater, and the result showed good agreement (*κ*, 0.82). Internal consistency was acceptable (*α*, 0.74). Confirmatory factor analysis showed all factor loadings of 10 essential quality indicators on 1 domain > 0.65 (Table 1).

Measurements of frequencies and the rangeof frequencies of leading behavior by each intensivist showed varied results and ranges per intensivist and per quality indicator (Table 2).

**Figure 1:**
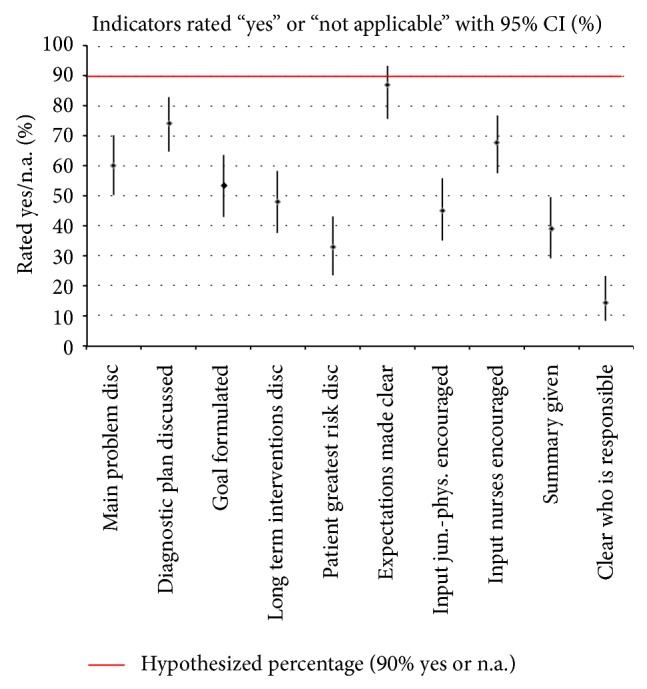
Results of the differences between the hypothesized and saturated model (with 95% confidence interval), with 99 patient presentations during 10 interdisciplinary rounds by 10 leading intensivists.

The differences between the hypothesized standard of 90% “yes” or “not applicable” cores and saturated results showed that 9 of 10 essential quality indicators were markedly rated lower than the hypothesized standard. Only 1 essential quality indicator (expectations made clear by consultants) was similar to the hypothesized standard of the 90% “yes” or “not applicable” scores (Figure 1).

## 4. Discussion

We have tested the measurement properties and implementation of a checklist aimed at assessing the leadership skills in leading IDRs in the ICU. The tests showed good interrater reliability and acceptable internal consistency. Confirmatory factor analysis showed all factor loadings on 1 domain. The checklist was applied to 99 patients' care plans which were discussed during IDRs in 4 adult ICUs led by 10 experienced intensivists. Frequency tests showed a wide range of “yes” and “no” responses among the intensivists.

Implementation of the checklist showed rating the essential indicators is appropriate for real-time assessment. We felt the use of videotaped IDRs was helpful in the process of evaluating and giving feedback to intensivists.

Strengths of this study included the use of a quantitative checklist to assess leadership skills while leading IDRs because this checklist identified issues that were not obvious to intensivists. Evaluation of the checklist during ward and staff meetings with all participants of the IDRs present demonstrated the attention of physicians may be dominated by choices which require immediate attention, such as ventilator settings, vasopressors, and imaging studies. Long term interventions and coordination are given little attention but are important. In addition, attention to the communication process is often taken for friendliness rather than insurance that appropriate technical choices are applied uniformly.

Limitations of this study include the performance of the study at a single centre, which may limit generalizability. In addition, in the beginning the intensivists were not inquisitive about the checklist because they considered their IDRs to be adequately performed and they were surprised by the results of the study. They assumed that they had discussed all relevant indicators in the same way. Familiarity with the checklist may have generated other outcomes. Furthermore, there was no assessment of the scores for predictive value for any type of patient outcome, such as length of stay.

The study has clinical relevance because adequate leadership skills may improve patient care. Positive associations were found between adequate interdisciplinary communication of leaders and satisfaction with quality of care and the ethical climate [37].

In the present study, large differences in the way senior physicians led IDRs became apparent, such as different manners of determining and/or communicating the patients' plan of care. This can cause confusion and conflicts among team members.

Although beyond the scope of our study, the varied performances of senior physicians while leading IDRs showed that the current way of learning to lead IDRs by role modeling may be an ineffective way. This is because many physicians may assimilate leadership techniques that are inadequate. The checklist may provide feedback for the leading physicians, the ICU team members, and management to guide individual leading skills and team leading skills, and this may improve the potential for developing appropriate treatment plans for the ICU patient.

Checklists are considered to be useful to structure the interdisciplinary communication process in complex and dynamic situations in the ICU, such as IDRs, in order to engage in a necessary strategy. This strategy is otherwise complicated because of diversity of perceptions, educational backgrounds, and responsibilities of team members and consultants [5, 38–40].

However, despite the corpus of evidence regarding the benefits of checklists, medicine remains slow in broadly adopting them into practice [23]. Therefore, before, during, and after implementation of this checklist, it remains important for identifying and mitigating local team barriers to complete checklist items. This can be effectuated by training sessions. During these (leadership) training sessions, it could be helpful to provide feedback by reviewing fragments of videotaped IDRs to improve awareness and to diminish the resistance of using checklists [32].

In the present study, the male to female ratio (9 : 1) may have skewed the results. Leadership behavior may be affected by gender and personality [41, 42]. During resuscitation tests female students showed less leadership behavior and had less hands-on time than male students. However, male providers had less leadership skill when tasks required complex social interactions, which required more relationship-oriented (female) leadership, in accordance with gender stereotypes [41, 42]. The effect of gender on leading IDRs and on leadership in general needs further study.

Future studies may evaluate the effect of using this checklist on the predictive value for outcomes such as staff, patient, or family satisfaction or clinical outcomes. In addition, future research may evaluate the extent to which scores improve when physicians were given the checklist to guide their meeting, and physicians may use the checklist as a self-assessment checklist at the end of the IDR. It may be necessary to repeat the present study in other health care settings than the ICU to further develop the checklist and establish generalizability.

## 5. Conclusion

The novel checklist which assesses the leadership skills of intensivists while leading IDRs in the ICU demonstrates good reliability and acceptable internal consistency. Assessing leadership skills of intensivists with the checklist showed varied ranges per intensivist and per quality indicator. Future work could be aimed at comparing the findings of the previously executed intervention study, in which the intervention group received a one-day training to improve their leading skills, with a new study in which controls use this novel checklist to evaluate themselves while leading IDRs [32]. It would be interesting to use this checklist on the predictive value for outcomes such as staff, patient, and family satisfaction or clinical outcomes.

## Figures and Tables

**Table 1:** Definitions of the 10 essential quality indicators of the checklist^*^.

Patient plan of care	
(1) Main problem discussed (0.917)^†^	
Verbal identification of the (provisional) main problem, according to patient's response to treatment, or same as indication(s) for admission to the ICU	
(2) Diagnostic plan discussed (0.897)	
To discuss specific activities (laboratory tests, computed tomography scans, radiographs, or consults with other consultants) for the purpose of determining diagnosis or excluding specific problems or complications	
(3) Provisional goal formulated (0.897)	
What must be done to get this patient to the next level of care or discharged from the ICU?	
(4) Long-term therapeutic items (>16 h) discussed (0.797)	
(5) Patient greatest risk discussed (0.668)	
The risk of a widespread or serious complication that can occur because of factors associated with the patient, therapy, or stay in the ICU, or same as indication(s) for admission of patient to the ICU	

Process	

(6) Expectations made clear by consultants (0.762)	
Consultant gives explanation, advice, or justification of specific therapeutic issues related to the patient	
(7) Input of junior physicians encouraged (0.710)	
Junior physicians have an opportunity to speak	
(8) Input of nurses encouraged (0.732)	
Nurses have an opportunity to speak	
(9) Summary given (0.867)	
Overview of patient's treatment plan is given: diagnoses, goals, therapy, priority, and identification of responsible providers when appropriate, the summary includes diagnostic plan	
(10) It is clear who is responsible for performing tasks (0.710)	
Core duties for team members are discussed tasks are cross-checked to ensure a shared understanding	

^*^Descriptions of each quality indicator were outlined in a manual for users. ICU: intensive care unit. ^†^Numbers in parentheses were the results of a confirmative factor analysis that found all factor loadings of 10 essential quality indicators on 1 domain.

**Table 2:** Implementation of the checklist of 10 essential indicators in clinical scenarios in the intensive care unit^*^.

Essential quality indicator	No (%)	Doubt (%)	Yes (%)	Not applicable (%)
*Domain “patient plan of care” *				
(1) Main problem discussed	21 (21)	19 (19)	59 (60)	—
(2) Diagnostic plan discussed	23 (23)	3 (3)	66 (67)	7 (7)
(3) Provisional goal formulated	24 (24)	23 (23)	52 (53)	—
(4) Long term interventions (>16 h) discussed	43 (43)	9 (9)	46 (47)	1 (1)
(5) Patient greatest risk discussed	59 (60)	8 (8)	32 (32)	0 (0)
*Domain “Process” *				
(6) Expectations made clear by consultants	14 (14)	0 (0)	85 (85)	0 (0)
(7) Input of junior physicians encouraged	27 (27)	28 (28)	41 (41)	3 (3)
(8) Input of nurses encouraged	17 (17)	16 (16)	66 (67)	0 (0)
(9) Summary given	49 (50)	12 (12)	38 (38)	—
(10) It is clear who is responsible for performing tasks	77 (78)	8 (8)	14 (14)	—

^*^ *N* = 99 patient presentations in 10 interdisciplinary rounds led by 10 senior physicians. Essential indicators of the checklist: each item was answered with either 1 (no), 2 (doubt), 3 (yes), or not applicable (except that there was no “not applicable” option for items 1, 3, 9, and 10. The data was reported as the number (%) of no, doubt, yes, or not applicable ratings).

## References

[1] Suter E., Goldman J., Martimianakis T., Chatalalsingh C., Dematteo D. J., Reeves S. (2013). The use of systems and organizational theories in the interprofessional field: findings from a scoping review. *Journal of Interprofessional Care*.

[2] Richardson J., West M. A., Cuthbertson B. H. (2010). Team working in intensive care: Current evidence and future endeavors. *Current Opinion in Critical Care*.

[3] Kim M. M., Barnato A. E., Angus D. C., Fleisher L. F., Kahn J. M. (2010). The effect of multidisciplinary care teams on intensive care unit mortality. *Archives of Internal Medicine*.

[4] Baggs J. G., Schmitt M. H., Mushlin A. I. (1999). Association between nurse-physician collaboration and patient outcomes in three intensive care units. *Critical Care Medicine*.

[5] Pronovost P., Berenholtz S., Dorman T., Lipsett P. A., Simmonds T., Haraden C. (2003). Improving communication in the ICU using daily goals. *Journal of Critical Care*.

[6] Manser T. (2009). Teamwork and patient safety in dynamic domains of healthcare: a review of the literature. *Acta Anaesthesiologica Scandinavica*.

[7] Salas E., Almeida S. A., Salisbury M. (2009). What are the critical success factors for team training in health care?. *Joint Commission Journal on Quality and Patient Safety*.

[8] Reader T. W., Flin R., Mearns K., Cuthbertson B. H. (2009). Developing a team performance framework for the intensive care unit. *Critical Care Medicine*.

[9] Azoulay É., Timsit J.-F., Sprung C. L. (2009). Prevalence and factors of intensive care unit conflicts: the conflicus study. *American Journal of Respiratory and Critical Care Medicine*.

[10] Dodek P. M., Raboud J. (2003). Explicit approach to rounds in an ICU improves communication and satisfaction of providers. *Intensive Care Medicine*.

[11] Ellrodt G., Glasener R., Cadorette B. (2007). Multidisciplinary rounds (MDR): an implementation system for sustained improvement in the American Heart Association's get with the guidelines program. *Critical Pathways in Cardiology*.

[12] Stockwell D. C., Slonim A. D., Pollack M. M. (2007). Physician team management affects goal achievement in the intensive care unit. *Pediatric Critical Care Medicine*.

[13] Have E. C. M. T., Nap R. E. (2014). Mutual agreement between providers in intensive care medicine on patient care after interdisciplinary rounds. *Journal of Intensive Care Medicine*.

[14] Suter E., Arndt J., Arthur N., Parboosingh J., Taylor E., Deutschlander S. (2009). Role understanding and effective communication as core competencies for collaborative practice. *Journal of Interprofessional Care*.

[15] Jain A. K., Thompson J. M., Chaudry J., McKenzie S., Schwartz R. W. (2008). High-performance teams for current and future physician leaders: an introduction. *Journal of Surgical Education*.

[16] Curtis J. R., Cook D. J., Wall R. J. (2006). Intensive care unit quality improvement: a 'how-to' guide for the interdisciplinary team. *Critical Care Medicine*.

[17] Guidet B., González-Romá V. (2011). Climate and cultural aspects in intensive care units. *Critical Care*.

[18] Rourke J., Frank J. R. (2005). Implementing the CanMEDS physician roles in rural specialist education: the multi-specialty community training network. *Education for health (Abingdon, England)*.

[19] Reeves S., Perrier L., Goldman J., Freeth D., Zwarenstein M. (2013). Interprofessional education: effects on professional practice and healthcare outcomes (update). *The Cochrane Database of Systematic Reviews*.

[20] Zwarenstein M., Goldman J., Reeves S. (2009). Interprofessional collaboration: effects of practice-based interventions on professional practice and healthcare outcomes. *Cochrane Database of Systematic Reviews*.

[21] Reeves S., Goldman J., Gilbert J. (2011). A scoping review to improve conceptual clarity of interprofessional interventions. *Journal of Interprofessional Care*.

[22] Winters B. D., Gurses A. P., Lehmann H., Sexton J. B., Rampersad C. J., Pronovost P. J. (2009). Clinical review: checklists—translating evidence into practice. *Critical Care*.

[23] Winters B. D., Aswani M. S., Pronovost P. J. (2011). Commentary: reducing diagnostic errors: another role for checklists?. *Academic Medicine*.

[24] Ten Have E. C. M., Hagedoorn M., Holman N. D., Nap R. E., Sanderman R., Tulleken J. E. (2013). Assessing the quality of interdisciplinary rounds in the intensive care unit. *Journal of Critical Care*.

[25] van Beuzekom M., Akerboom S. P., Boer F. (2007). Assessing system failures in operating rooms and intensive care units. *Quality and Safety in Health Care*.

[26] Boyle D. K., Kochinda C. (2004). Enhancing collaborative communication of nurse and physician leadership in two intensive care units. *Journal of Nursing Administration*.

[27] Reader T. W., Flin R., Cuthbertson B. H. (2011). Team leadership in the intensive care unit: the perspective of specialists. *Critical Care Medicine*.

[28] Pronovost P. J., Miller M. R., Wachter R. M., Meyer G. S. (2009). Perspective: physician leadership in quality. *Academic Medicine*.

[29] Pronovost PJ. (2012). Bridging the leadership development gap: recommendations for medical education. *Academic Medicine*.

[30] Ohlinger J., Brown M. S., Laudert S., Swanson S., Fofah O. (2003). Development of potentially better practices for the neonatal intensive care unit as a culture of collaboration: communication, accountability, respect, and empowerment. *Pediatrics*.

[31] Malling B., Mortensen L., Bonderup T., Scherpbier A., Ringsted C. (2009). Combining a leadership course and multi-source feedback has no effect on leadership skills of leaders in postgraduate medical education. An intervention study with a control group. *BMC Medical Education*.

[32] Ten Have E. C. M., Nap R. E., Tulleken J. E. (2013). Quality improvement of interdisciplinary rounds by leadership training based on essential quality indicators of the Interdisciplinary Rounds Assessment Scale. *Intensive Care Medicine*.

[33] Hawthorne G., Richardson J., Osborne R. (1999). The Assessment of Quality of Life (AQoL) instrument: a psychometric measure of health-related quality of life. *Quality of Life Research*.

[34] Cook D. A., Beckman T. J. (2006). Current concepts in validity and reliability for psychometric instruments: theory and application. *The American Journal of Medicine*.

[36] Randolph J. J. (2005). Free-marginal multirater kappa: An alternative to Fleiss' fixed-marginal multirater kappa. *ERIC Document Reproduction Service no.*.

[37] Le Blanc P. M., Schaufeli W. B., Salanova M., Llorens S., Nap R. E. (2010). Efficacy beliefs predict collaborative practice among intensive care unit nurses. *Journal of Advanced Nursing*.

[38] Simpson S. Q., Peterson D. A., O'Brien-Ladner A. R. (2007). Development and implementation of an ICU quality improvement checklist. *AACN Advanced Critical Care*.

[39] Agarwal S., Frankel L., Tourner S., McMillan A., Sharek P. J. (2008). Improving communication in a pediatric intensive care unit using daily patient goal sheets. *Journal of Critical Care*.

[40] Puntillo K. A., McAdam J. L. (2006). Communication between physicians and nurses as a target for improving end-of-life care in the intensive care unit: challenges and opportunities for moving forward. *Critical Care Medicine*.

[41] Hunziker S., Laschinger L., Portmann-Schwarz S., Semmer N. K., Tschan F., Marsch S. (2011). Perceived stress and team performance during a simulated resuscitation. *Intensive Care Medicine*.

[42] Streiff S., Tschan F., Hunziker S. (2011). Leadership in medical emergencies depends on gender and personality. *Simulation in Healthcare*.

